# Do Patients’ Psychosocial Characteristics Impact Antibiotic Prescription Rates?

**DOI:** 10.3390/antibiotics12061022

**Published:** 2023-06-07

**Authors:** Säde Stenlund, Louise C. Mâsse, David Stenlund, Lauri Sillanmäki, Kirstin C. Appelt, Heli Koivumaa-Honkanen, Päivi Rautava, Sakari Suominen, David M. Patrick

**Affiliations:** 1 School of Population and Public Health, University of British Columbia, Vancouver, BC V6T 1Z3, Canada; lmasse@bcchr.ubc.ca (L.C.M.); david.patrick@ubc.ca (D.M.P.); 2 BC Centre for Disease Control, Vancouver, BC V5Z 4R4, Canada; 3 Department of Public Health, University of Turku, 20014 Turku, Finlandrautava@utu.fi (P.R.); suominen@utu.fi (S.S.); 4 Research Services, Turku University Hospital, 20520 Turku, Finland; 5 BC Children’s Hospital Research Institute, University of British Columbia, Vancouver, BC V5Z 4H4, Canada; 6 Department of Mathematics, University of British Columbia, Vancouver, BC V6T 1Z2, Canada; stenlund@math.ubc.ca; 7 Faculty of Science and Engineering, Åbo Akademi University, 20500 Turku, Finland; 8 Department of Public Health, University of Helsinki, 00014 Helsinki, Finland; 9 Sauder School of Business, University of British Columbia, Vancouver, BC V6T 1Z2, Canada; kirstin.appelt@sauder.ubc.ca; 10 Institute of Clinical Medicine (Psychiatry), University of Eastern Finland, 70029 Kuopio, Finland; heli.koivumaa@uef.fi; 11 School of Health Sciences, University of Skövde, 54128 Skövde, Sweden

**Keywords:** antibiotic prescribing, antibiotic consumption, psychosocial, structural equation modeling, excess antibiotic use, antimicrobial stewardship

## Abstract

Previous research suggests that the characteristics of both patients and physicians can contribute to the overuse of antibiotics. Until now, patients’ psychosocial characteristics have not been widely explored as a potential contributor to the overuse of antibiotics. In this study, the relationship between a patient’s psychosocial characteristics (self-reported in postal surveys in 2003) and the number of antibiotics they were prescribed (recorded in Finnish national registry data between 2004–2006) were analyzed for 19,300 working-aged Finns. Psychosocial characteristics included life satisfaction, a sense of coherence, perceived stress, hostility, and optimism. In a structural equation model, patients’ adverse psychosocial characteristics were not related to increased antibiotic prescriptions in the subsequent three years. However, these characteristics were strongly associated with poor general health status, which in turn was associated with an increased number of subsequent antibiotic prescriptions. Furthermore, mediation analysis showed that individuals who used healthcare services more frequently also received more antibiotic prescriptions. The current study does not support the view that patients’ adverse psychosocial characteristics are related to an increased number of antibiotic prescriptions. This could encourage physicians to actively discuss treatment options with their patients.

## 1. Introduction

Antimicrobial resistance is one of the leading health threats of the 21st century and requires global action [1]. One of five key strategies identified to address the problem is the avoidance of antibiotic prescriptions lacking a clear medical indication [2]. A significant proportion of prescribed antibiotics is not medically necessary, with a rate as high as one-third reported from the US [3]. Optimizing antibiotic prescribing is complex for physicians [4], who report that factors beyond the patient’s clinical status influence their decision to prescribe [5]. Furthermore, previous research indicates that antibiotic prescribing is influenced by both physicians’ and patients’ non-medical characteristics.

For physicians, impactful factors include time pressure, decision fatigue, ignorance of medical guidelines, and fear of accusations or adverse consequences related to under-treatment [3,6,7,8,9,10]. Physicians’ willingness to prescribe may also be impacted by unfounded assumptions that their patients prefer to be prescribed antibiotics [11]. Even the personal characteristics of physicians can affect their decisions. Physicians who are men, older, or immigrants prescribe antibiotics more often [12]. Physicians may also prefer to trust their previous experience and familiarity with antibiotic prescribing rather than change their prescribing habits [13,14].

Similarly, patients’ personal characteristics, cultural norms, and perceptions can affect antibiotic consumption [8]. For example, white patients are prescribed antibiotics more often [12]. In Korea, appropriate antibiotic consumption is correlated with being male, being married, and having better knowledge [15]. Better knowledge is, in turn, related to being younger, having more media exposure, and having a college or healthcare-related education. Lastly, if patients believe that antibiotics are effective against viruses, they are more likely to expect antibiotics [16]; however, there is consistent observation that patient demand for antibiotics has decreased [17].

Thus, both patients and physicians can contribute to excess antibiotic use. This study was conceived to further explore the influence of non-medical factors on antibiotic prescribing, with a focus on patients’ psychosocial characteristics, which have been sparsely studied. A patient’s adverse psychosocial characteristics could increase the physician’s perception that the patient is demanding antibiotics or decrease the physician’s willingness to find an alternative resolution for the appointment. The present observational study explores whether patients’ self-reported psychosocial characteristics, specifically their sense of coherence, hostility, optimism, stress, and life satisfaction, predict how often they are prescribed antibiotics. We hypothesize that patients with adverse psychosocial characteristics might receive more antibiotics as they may appear more demanding during their appointments.

## 2. Materials and Methods

### 2.1. Participants

**Figure 1:**
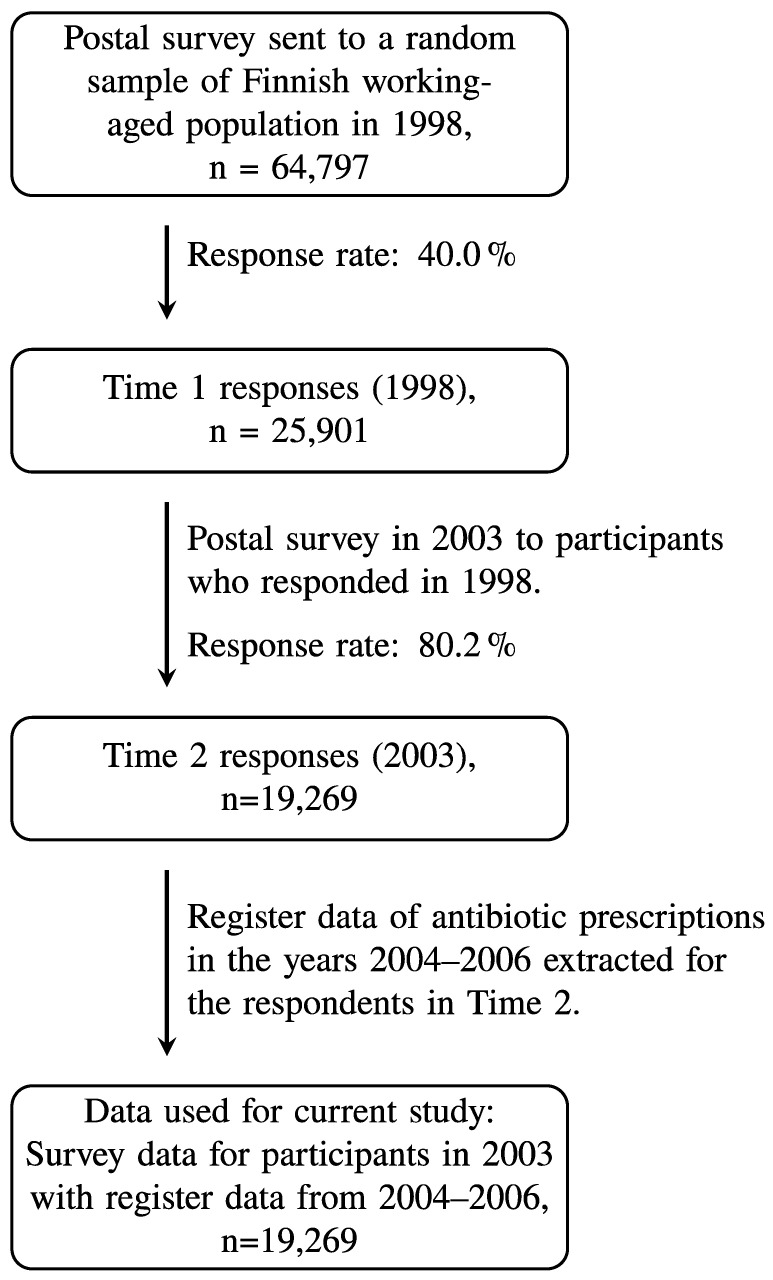
Flow chart of the study population.

The present study used patient characteristics that were self-reported in postal surveys as part of the 2003 Health and Social Support (HeSSup) study. These survey responses were linked to the Social Insurance Institution of Finland’s (KELA) national registry data on antibiotics purchases in the years 2004–2006. The 1998 HeSSup study is based on a representative random sample of working-aged Finns (n = 64,797, response rate 40%) in the age brackets 20–24, 30–34, 40–44, and 50–54 years. The current study used data from the 2003 HeSSup follow-up survey (n = 19,269, 80% response rate from previous respondents; for details, see Figure 1), which surveyed a broader set of psychological characteristics. The original HeSSup study was approved by the concurrent joint Ethical Committee of the University of Turku and the Turku University Hospital. Respondents gave their signed consent for a prospective follow-up, including certain national health registry data. The data were anonymized before analysis and neither the personal integrity nor privacy of the participants was violated.

### 2.2. Measures

Patients’ self-reported *psychosocial characteristics* included five validated scales (details below), which were all coded such that higher values indicate more adverse characteristics. The total score of a scale was not computed if more than 25% of its items were missing, except for the sense of coherence scale, where at least half of the items of each subcomponent were required for the total score to be computed.

The *Cook–Medley Hostility Scale* measures beliefs and behavior towards other people [18] (Cronbach’s alpha = 0.84). It was adjusted to include eight items [19], such as “I think most people would lie to get ahead” and “It is safer to trust nobody”. Each item was scored 1–5 (“completely agree”—“completely disagree”). The total score 8–40 was divided into quartiles for this study.

The four-item *Life Satisfaction Scale* (Cronbach’s alpha = 0.78) measures happiness, easiness, interestingness in life, and perceived loneliness [20,21]. The first three questions start with “Do you feel your life at present is…” whereas the fourth question starts with “Do you feel that at the present you are…” Each item is scored 1–5. The sum score (range 4–20) was divided into tertiles: 4–6 (satisfied), 7–11 (intermediate), and 12–20 (dissatisfied).

The *Life Orientation Test* (Cronbach’s alpha = 0.78) assesses optimism and pessimism [22]. Six items (score 1–5), such as “In uncertain times, I usually expect the best” resulted in a total score (range 6–30), which was split into tertiles: <20; 20–24; >24.

The *Sense of Coherence Scale* (Cronbach’s alpha = 0.85) is measured using Antonovsky’s 13-item scale [23]. The scale contains three subcomponents on how manageable (four questions), comprehensible (five questions), and meaningful (four questions) life feels [24], including questions, such as “Do you have the feeling that you don’t really care about what goes on around you?” (meaningfulness) and “Has it happened in the past that you were surprised by the behavior of people whom you thought you knew well?” (comprehensibility). Each item was scored on a seven-point Likert-type scale, and the sum score (range 13–91) had a cut-off at the lower and upper quartiles.

The *Reeder Stress Inventory* assessed perceived stress through four items (score 1–5), such as “I am, in general, usually tense and nervous.” [25] (Cronbach’s alpha = 0.77). The total score of 4–20 was divided into tertiles: <13, 13–16, >16.

To describe overall *health status*, the present study used three health measures. The number of self-reported chronic diseases from a pre-defined list of 35 conditions (for details, see [26]) was categorized into 0, 1, 2, and 3 or more chronic conditions. The number of reported medicines (in 13 categories) used for more than six months was grouped into 0, 1, and 2 or more regular medicines. NYHA (New York Heart Association) symptom classification for heart failure (0–4) was used to describe the severity of current symptoms.

Furthermore, the *tendency to use healthcare* was categorized based on self-reported visits (0, 1, 2–4, 5 or more) to the following types of physicians during the previous year: public primary care physician, occupational care physician, a physician at a hospital, and private physician. The responses were categorized as follows: (1) no visits to any doctor, (2) one visit to a doctor, (3) two to four visits to a single type of doctor or single visits to multiple types of doctors, and (4) more than five visits to a specific type or several visits to multiple types of doctors.

*Sociodemographic characteristics* include age (in 2003) in four groups: (1) 25–29 years, (2) 35–39 years, (3) 45–49, and (4) 55–59; registry-based gender in two categories, male and female (in 1998); and education (in 2003) on four levels: (1) no professional education, (2) vocational course/school/apprenticeship contract, (3) college, and (4) university degree/university of applied sciences.

Antibiotic use and the underlying health condition could affect both stress and life satisfaction. Therefore, psychosocial characteristics were self-reported in 2003, whereas antibiotic prescriptions were measured from 1 January 2004 to 31 December 2006. The number of antibiotics prescribed was obtained from the registry data of the Social Insurance Institution of Finland, which records all systemic antibiotics purchased by prescription in Finland. The purchases in the Anatomical Chemical Code (ATC) class J01 (antibacterial for systemic use) were extracted, categorized into five classes (0, 1, 2, 3, and 4 or more prescriptions), and linked with the participants’ survey data.

### 2.3. Statistical Analysis

Cronbach’s alphas were computed for the psychosocial measures (presented in the methods section along with the scales) with SAS 9.4 TS1M5 (SAS Institute Inc., Cary, NC, USA, 2016). Structural equation modeling (SEM) was used to explore the potential association between psychosocial factors, antibiotics purchased, health status, and tendency to use healthcare. The model was initially run on half of the data and cross-validated in the other half. As the findings were replicated in both datasets, the results show the findings of the whole dataset. The SEM analyses were conducted in Mplus software, version 7.4 [27]. Overall model fit was assessed using a number of indices because there are no agreed-upon standards [28]. Following Hu and Bentler’s recommendation, the fit of the models was evaluated using the following indices (levels of acceptable fit): the Comparative Fit Index (CFI > 0.95), the Tucker–Lewis Index (TLI > 0.95), Standardized Root Mean Square Residual (SRMR < 0.08), and the Root Mean Square Error of Approximation (RMSEA upper 95% confidence interval less than 0.08). Chi-square values (χ^2^) were not used for model fit estimation because a good fit can be hard to achieve in large samples [29].

In preparation for the SEM analyses, confirmatory factor analysis was first performed separately to examine the latent psychosocial factors (hostility, life satisfaction, optimism, sense of coherence, and stress) and the latent health status factor (self-reported chronic diseases, regular medications, and NYHA symptoms), then together to determine whether a two-factor structure was supported in the dataset. The latent factor for health status had an excellent fit (CFI = 1.0; TLI = 1.0; and RMSEA < 0.001; χ^2^ = 2910 (df = 3), *p* < 0.001). The latent factor for the five psychosocial factors had an adequate fit initially, but hostility and optimism had unexplained residuals with a sense of coherence. Given that hostility and optimism were theoretically associated with a sense of coherence, these two scales were dropped, which resulted in an adequate fit (CFI = 1.0; TLI = 1.0; and RMSEA < 0.001; χ^2^ = 5040 (df = 3), *p* < 0.001). The two-factor model showed adequate fit (CFI = 0.967; TLI = 0.938; and RMSEA = 0.06 (95% CI = 0.054–0.066); χ^2^ = 8680 (df = 15), *p* < 0.001). In the SEM analyses, the effect of the latent psychosocial factor and health status factor on subsequent antibiotic prescriptions was first explored by adding the number of antibiotics in 2004–2006 as an outcome. Then, the potentially mediating role of healthcare use was further explored. In these analyses, the following socio-economic covariates were entered into the model: age, gender, and education. However, because the covariates had a negative impact on the overall fit of the model, they were omitted.

## 3. Results

The demographic characteristics of the study population are presented in Table 1. Of the respondents, 61% were female. The four age groups were fairly equally represented, with a slightly higher percentage in the oldest (55–59 years, 27.6%) and a slightly lower percentage in the lower middle group (35–39 years, 22.6%). Both the lowest and highest education groups were somewhat less represented (12% and 19%, respectively). Most participants had no regular medications (69%) and no NYHA classified symptoms (66%), but a total of 81% had one or more chronic diseases. Most participants reported intermediate levels on the psychosocial measures: life satisfaction (56%), sense of coherence (51%), and stress (49%). According to registry data, almost half (49%, n = 9674) of the study population did not receive any antibiotics during the measurement period. One prescription was received by 22% (n = 4323), two by 12% (n = 2408), three by 7% (n = 1283), and four or more prescriptions were received by 10% (n = 1941) of the studied population.

**Figure 2:**
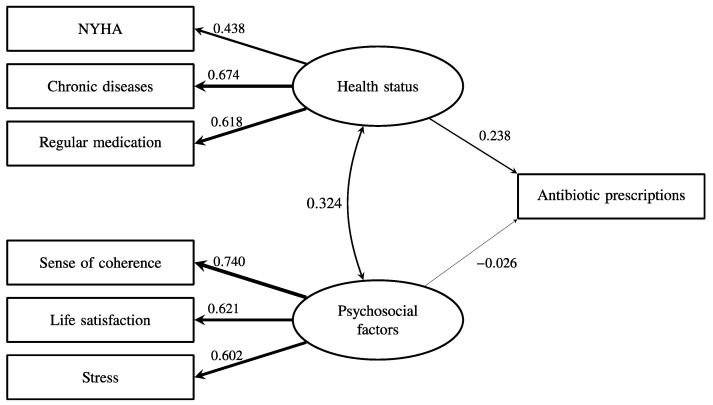
The association between health status, psychosocial factors, and the subsequent frequency of antibiotic prescription. The numbers and relative line widths indicate the effect sizes of the pathways in the structural equation model.

**Figure 3:**
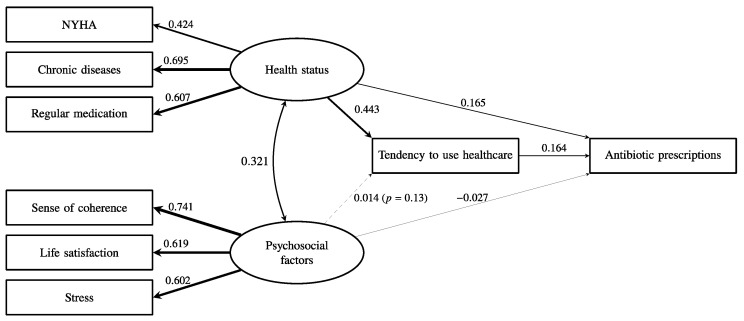
The association between health status, psychosocial factors, and the subsequent frequency of antibiotic prescription with the mediation by a tendency to use healthcare. The indirect pathway from health status to prescription of antibiotics was significant (β = 0.073, *p* < *0*.001). The numbers and relative line widths indicate the effect sizes of the pathways in the structural equation model.

The latent psychosocial factor had a very low path coefficient with subsequent antibiotic prescriptions (β = 0.055, *p* < 0.001). As shown in Figure 2, health status was significantly associated with more antibiotic prescriptions (β = 0.238, *p* < 0.001), but psychosocial factors (β = −0.026, *p* < 0.001) were not associated with more antibiotic prescriptions even though psychosocial factors were significantly associated with health status (β = 0.324, *p* < 0.001). As shown in Figure 3, the tendency to use healthcare partially mediated the effect of health status on antibiotic prescriptions because the direct effect of health status on antibiotic prescriptions remained significant (β = 0.165, *p* < 0.001), and the indirect effect of health status on antibiotic prescriptions via healthcare use was also significant (β = 0.073, *p* < 0.001). Overall, this model explained 20% of the variance in antibiotic prescriptions. The fit of the final model in Figure 3 was acceptable (CFI = 0.97, TLI = 0.95, SRMR = 0.029, RMSEA = 0.046 (95% CI = 0.043–0.049)).

## 4. Discussion

The present study of 19,300 working-aged Finns was conducted to explore the influence of non-medical factors on antibiotic prescriptions. It did not provide evidence that patients’ psychosocial characteristics increase the likelihood of receiving antibiotics, even if these psychosocial characteristics did correlate strongly with health status, which was a moderate predictor of antibiotic prescriptions. In addition, the tendency to use healthcare shows partial mediation from health status to antibiotic prescriptions.

The study design could not identify whether the antibiotic prescriptions were appropriate. However, it provides some evidence that adverse psychosocial characteristics in patients are not associated with an increased number of prescriptions. This is in line with previous studies suggesting that patient-related factors may not be the major driver of unnecessary antibiotic prescriptions [3,6,7,8,9,11]. However, physicians’ perception of patients’ preferences for antibiotics do contribute to excess prescriptions [8,11], and physicians perceive these as being the most important reason for excess prescribing [30]. The current results were obtained from a large sample of patients and take into account patients’ life satisfaction, optimism, perceived stress, hostility, sense of coherence, and overall health status.

Based on previous research and the current study, physicians could be encouraged to reflect critically on their prescription habits and their assumptions about patients. The evidence suggests that patient-related factors might not be as strong predictors of antibiotic prescriptions as physicians perceive them to be [11]. In the current study, these characteristics do correlate with worse health status, which moderately associates with subsequent antibiotic prescriptions. This may indicate that patients with poorer health receive more antibiotics and the care they presumably need. Based on these results, rather than assuming that patients are hostile toward non-antibiotic treatment options, physicians could be more willing to engage in shared decision-making to reduce unnecessary prescribing [31]. Therefore, the results could increase physicians’ confidence in using tools that suggest alternative treatments instead of antibiotics. One such tested strategy is suggesting alternative treatments with the help of a computerized clinical decision tool [32]. Similarly, these results could be considered when applying behavioral models to design other strategies to address prescribing [32,33,34]. These findings could also be valuable in medical education, supporting physicians in developing good prescribing practices. Improving prescribing is important because appropriate prescribing is a key strategy to combat antimicrobial resistance [2].

Around the turn of the millennium, Finland had one of the most extensive antibiotic stewardship programs in the world [35]. Through its collaboration with healthcare centers, public campaigns, and increased emphasis on guidelines, antibiotic prescription rates decreased considerably. As a result, the current research period represents an era when guidelines and appropriate prescribing practices were strongly emphasized in Finland. In many other countries, an emphasis on appropriate prescribing was established much later. For example, the World Health Organization called for national antimicrobial stewardship action plans in 2015 [36]. Therefore, although the current data is from almost two decades ago, it comes from an era of active antibiotic stewardship in Finland. Since the study period, awareness of antimicrobial resistance and the harmful effects of antibiotics have further increased, and patient preferences for being prescribed antibiotics have decreased [17]. However, physicians still report that they see the patient’s demands as the major driver of unnecessary prescribing [30]. Therefore, this study is important in showing that psychosocial factors in patients may not be causing an increased number of antibiotics received.

The present study uses a large population-based sample to explore the relationship between registry-based antibiotic prescriptions and self-reported psychosocial and health status measures. The Finnish national registry data includes all systemic antibiotics purchased in Finland and linking it to the HeSSup survey data provides a unique opportunity to study these relationships. The psychosocial characteristics were chosen based on survey items, but they were also able to provide specific perspectives on personality. SEM enabled the inclusion of various types of psychosocial and health factors, providing a better reflection of real life instead of relying on the choice of a specific measure. Nevertheless, psychosocial characteristics are best measured by self-reports providing a reflection of a person’s perception of oneself and one’s life. Perceived stress could be considered a momentary experience rather than a personal characteristic and, therefore, not of relevance for future outcomes. However, it can reflect how an individual perceives oneself in different life situations. Perceived stress fitted well in the confirmatory factor analysis along with the other more stable psychosocial characteristics. The psychosocial and health factors were measured at a separate time point compared to antibiotic prescriptions, which reduced the risk of an acute condition causing both psychosocial distress and antibiotic prescriptions. To reduce the effect of chronic conditions on both psychosocial distress and antibiotic prescriptions, we included health status in the structural equation model.

The study did not provide evidence that psychosocial characteristics increased the number of received antibiotics. The good fit of the final model and the strong associations between psychosocial characteristics, health status, and doctor visits indicate the validity of the data and the model, which increases the credibility of the results.

Although the study design was unique in combining survey and registry data to explore determinants of antibiotic prescribing, the pre-existing data caused limitations for the study. The study was purely observational and based on survey and registry data from almost two decades ago. Only a limited number of factors that might affect prescriptions and the interaction between physician and patient were included in the study. Appointment-specific measures, such as physicians’ problems communicating with patients or perceived patient hostility, were not explored. Some patients might also occasionally be very determined to receive antibiotics, which might lead to them exaggerating their symptoms. Such dynamics were outside the scope of our study. Other relevant factors, such as the deprivation of the patient, could not be measured by validated scales due to limitations of the data. Furthermore, the study could not identify if the received prescriptions were inappropriate. After the study period, antimicrobial resistance gained more attention globally, and patients were better educated about the risks of taking antibiotics when they are not needed, though these had already been emphasized actively in Finland before the study period. A certain degree of attrition has resulted in the overrepresentation of women, which is typical for postal health surveys [37]. However, the study population has been determined to include a representative sample of the Finnish general population [38], and the results can, therefore, be generalized to the Finnish population. Physicians’ practices, culture, and patient characteristics vary between countries, but these results can be generalized with some caution to countries where the patient–doctor relationship has become less authoritarian in recent decades and where antibiotic stewardship efforts have been or are being established.

This exploration opens multiple directions for further study. The association between patients’ psychosocial characteristics and antibiotic prescriptions should be studied using more recent data to strengthen the evidence. A wider set of patient factors, such as deprivation, should also be explored as potential drivers of increased antibiotic prescriptions. Factors influencing patients’ prescription preferences and their relation to psychosocial characteristics could also be explored, such as the experience of previous prescriptions or consultations [39]. Furthermore, being able to identify inappropriate prescribing is important when studying the effect of psychosocial factors on unnecessary prescribing. Additionally, studying the dynamics of real-life appointments through observation could provide a deeper understanding of how the patient’s psychosocial characteristics contribute to the outcome of the appointment. Finally, behavioral models could point toward additional non-medical factors that can affect antibiotic prescribing.

## 5. Conclusions

The current study does not provide evidence that psychosocial characteristics, including life satisfaction, sense of coherence, and perceived stress, are associated with a higher number of antibiotic prescriptions. However, a patient’s worse health status is associated with a higher number of prescriptions. This is in line with previous results stating that physician-related factors are more significant in predicting antibiotic prescriptions than patient-related factors and can encourage physicians to actively discuss treatment options with their patients.

## Figures and Tables

**Table 1:** Number of antibiotic prescriptions from registry data and self-reported tendency to use healthcare according to self-reported sociodemographic characteristics, health variables, and psychosocial characteristics. Missing covariate values result in a slightly different total number of cases included in different subgroups (19,363–19,626).

Variable	Categories	Share of the Study Population, % (n)	Number of Antibiotic Prescriptions 2004–2006, Mean (SD)	Tendency to Use Healthcare (SD)
Whole sample		19,626 (100%)	2.48 (2.3)	1.74 (1.0)
Age (2003)	25–29	4889 (24.9%)	2.38 (1.93)	1.69 (0.99)
	35–39	4437 (22.6%)	2.51 (2.28)	1.71 (1.00)
	45–49	4889 (24.9%)	2.48 (2.21)	1.72 (1.00)
	55–59	5411 (27.6%)	2.56 (2.72)	1.83 (1.00)
Gender	Male	7568 (38.6%)	2.25 (2.13)	1.58 (1.03)
	Female	12,058 (61.4%)	2.59 (2.39)	1.84 (0.95)
Educational level (2003)	No professional education	2431 (12.4%)	2.50 (2.35)	1.83 (0.99)
	Vocational school	5944 (30.3%)	2.47 (2.35)	1.75 (1.00)
	College	7497 (38.2%)	2.51 (2.29)	1.74 (0.98)
	University	3635 (18.5%)	2.37 (2.20)	1.66 (0.97)
Number of chronic diseases (2003)	0	3720 (19.0%)	2.01 (1.59)	1.29 (0.98)
	1	4637 (23.6%)	2.19 (1.91)	1.51 (0.97)
	2	3953 (20.1%)	2.39 (2.01)	1.73 (0.95)
	3 more	7194 (36.6%)	2.82 (2.73)	2.13 (0.87)
Number of regular medications (2003)	0	13,566 (69.1%)	2.26 (1.88)	1.58 (1.00)
	1	3757 (19.1%)	2.71 (2.46)	2.00 (0.88)
	2 or more	2223 (11.3%)	3.11 (3.41)	2.30 (0.78)
NYHA classification	0	12,951 (66.0%)	2.33 (2.02)	1.64 (0.99)
	1	4913 (25.0%)	2.46 (2.34)	1.87 (0.96)
	2	1207 (6.1%)	2.98 (3.17)	2.19 (0.88)
	3	176 (0.9%)	3.55 (4.00)	2.26 (0.90)
	4	230 (1.2%)	3.82 (4.11)	2.26 (0.86)
Life satisfaction (2003)	Satisfied (score 4–6)	4732 (24.1%)	2.45 (2.14)	1.61 (0.97)
	Intermediate (7–11)	11,026 (56.2%)	2.44 (2.29)	1.73 (0.98)
	Dissatisfied (12–20)	3674 (18.7%)	2.60 (2.50)	1.90 (1.00)
Sense of coherence	High coherence	4763 (24.3%)	2.37 (2.12)	1.56 (0.98)
	Intermediate	9904 (50.5%)	2.47 (2.37)	1.73 (0.98)
	Low coherence	4875 (24.8%)	2.59 (2.31)	1.92 (0.98)
Experienced stress	Little stress (score > 16)	5309 (27.0%)	2.43 (2.18)	1.63 (0.99)
	Intermediate (13–16)	9635 (49.1%)	2.43 (2.30)	1.72 (0.98)
	Much stress (score < 13)	4419 (22.5%)	2.60 (2.41)	1.93 (0.97)

## Data Availability

The dataset analyzed is not publicly available due to the study data containing variables of personal and sensitive nature and hence, due to the present legislation of Finland and the General Data Protection Regulation (GDPR) of the European Union, cannot be made openly accessible inside or outside Finland. On reasonable requests, the data is available from the authors in special cases.
